# Supplementary material for: sCentInDB: a database of essential oil chemical profiles of Indian medicinal plants
Source: Mol Divers. 2025 May 9;30(1):789–807. doi: 10.1007/s11030-025-11215-5 (PMC12926241; doi:10.1007/s11030-025-11215-5)
Supplement: Supplementary file 1 — Supplementary file1 (PDF 495 KB) [file 11030_2025_11215_MOESM1_ESM.pdf]

Supplementary Figures S1-S2

for

sCentInDB: A database of essential oil chemical profiles of Indian medicinal plants

Shanmuga Priya Baskaran[a,b], Geetha Ranganathan[a], Ajaya Kumar Sahoo[a,b], Kishan Kumar[a], Jayalakshmi Amaresan[a], Kundhanathan Ramesh[a], R.P. Vivek-Ananth[a,b,†], Areejit Samal[a,b,*]

[a] *The Institute of Mathematical Sciences (IMSc), Chennai 600113, India*

[b] *Homi Bhabha National Institute (HBNI), Mumbai 400094, India*

[†] *Present Address: Buck Institute for Research on Aging, Novato, CA, United States*

[*]Corresponding author: asamal@imsc.res.in

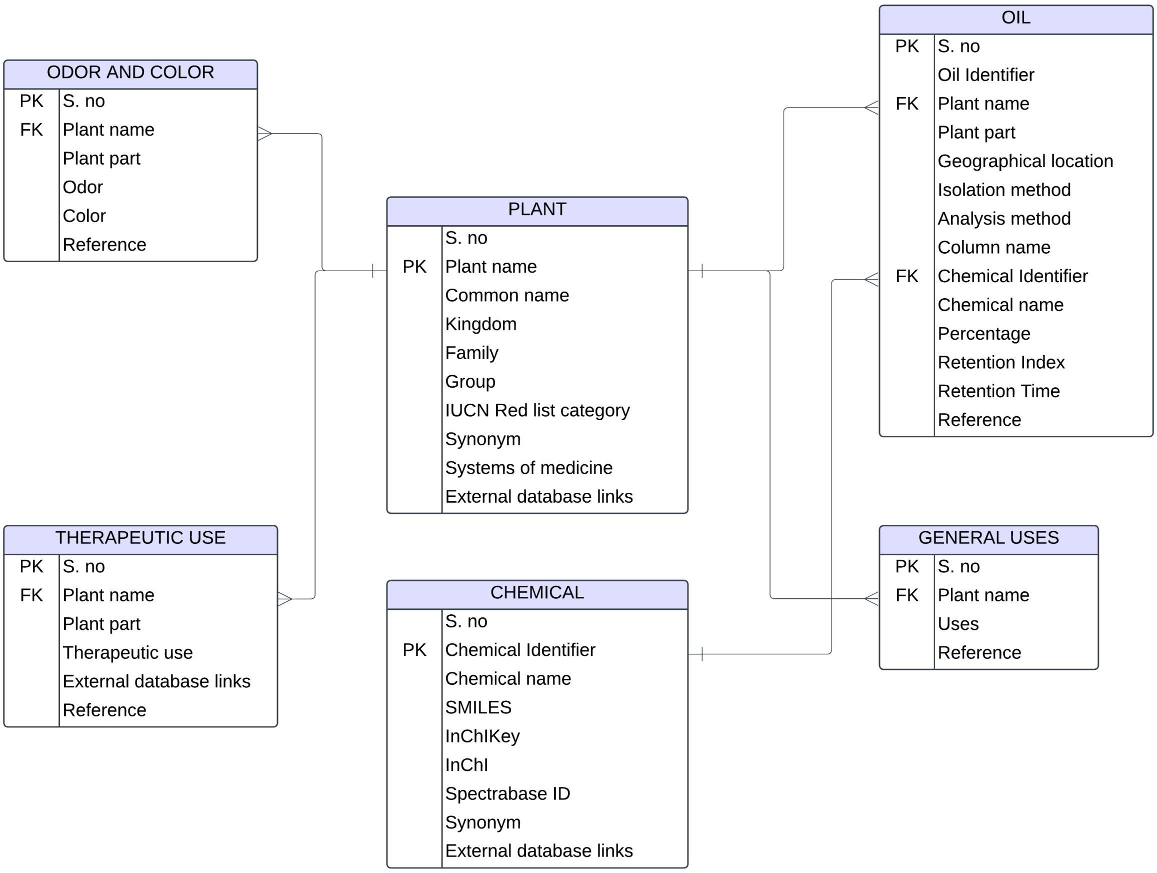

Figure S1: The Entity-Relationship Diagram (ERD) for the sCentInDB database illustrating the relationships between various attributes across different entities. The Plant table serves as the primary entity, establishing connections with other entities such as Oil, Odor and Color, Therapeutic use, and General Uses. Additionally, the Chemical entity is connected with the Oil entity through the Chemical Identifier attribute.

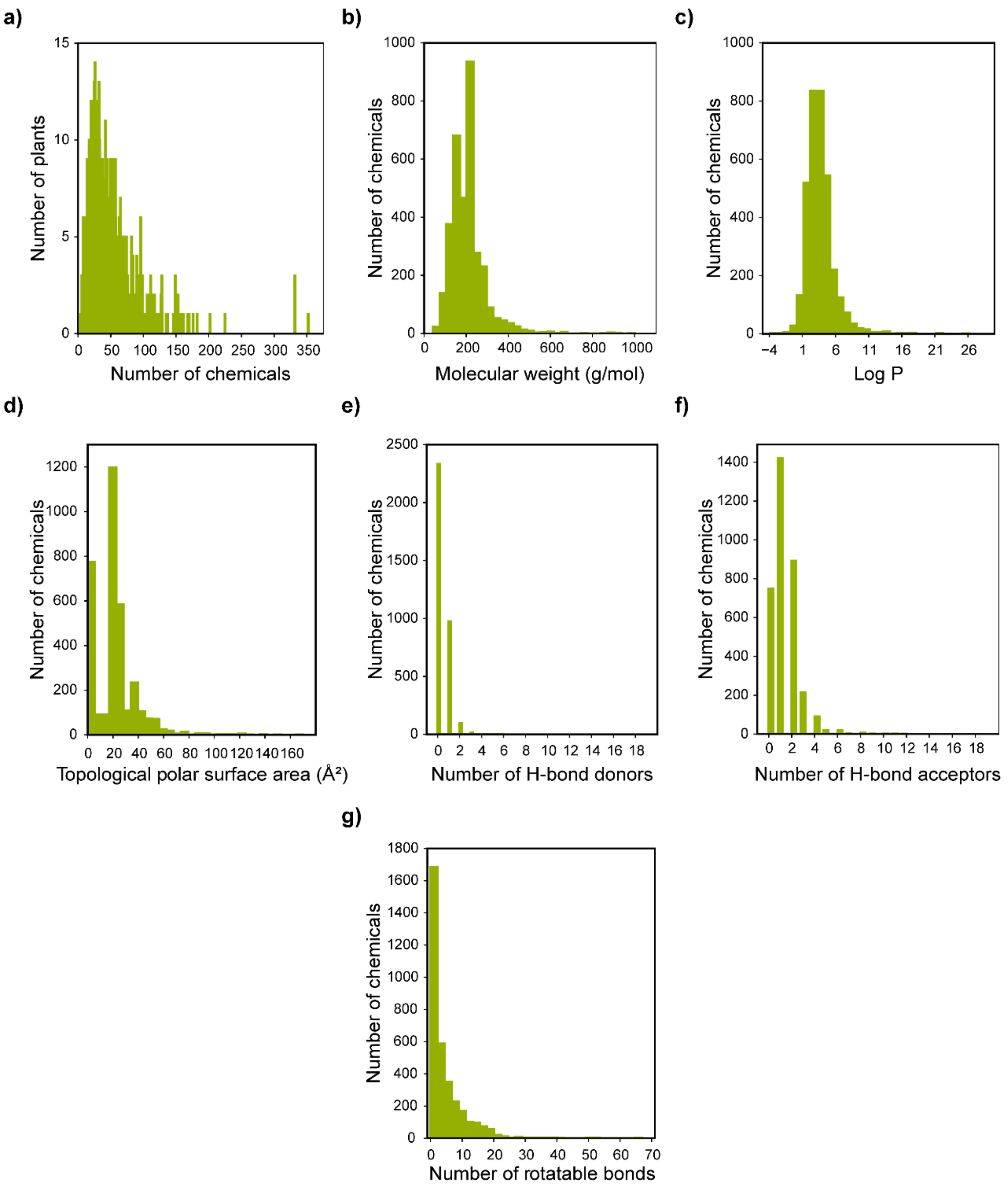

Figure S2: Basic statistics and physicochemical properties of essential oil chemicals in sCentInDB. **(a)** Distribution of Indian medicinal plants based on the number of associated chemicals. Distribution of six important physicochemical properties for the 3420 chemicals in sCentInDB: **(b)** molecular weight in g/mol, **(c)** Log P, **(d)** topological polar surface area in Å^2, **(e)** number of hydrogen bond donors, **(f)** number of hydrogen bond acceptors, and **(g)** number of rotatable bonds.
